# Centromere Protein F in Tumor Biology: Cancer's Achilles Heel

**DOI:** 10.1002/cam4.70949

**Published:** 2025-05-19

**Authors:** Zitong Wan, Miaomiao Wen, Chunlong Zheng, Ying Sun, Yinxi Zhou, Yahui Tian, Shaowei Xin, Xuejiao Wang, Xiaohong Ji, Jie Yang, Yanlu Xiong, Yong Han

**Affiliations:** ^1^ Department of Thoracic Surgery, Air Force Medical Center Fourth Military Medical University Beijing China; ^2^ Department of Thoracic Surgery, Tangdu Hospital Fourth Military Medical University Xi'an China; ^3^ College of Life Sciences Northwestern University Xi'an China; ^4^ Department of Thoracic Surgery 962 Hospital of the Joint Logistics Support Force Harbin China; ^5^ Innovation Center for Advanced Medicine, Tangdu Hospital Fourth Military Medical University Xi'an China; ^6^ Department of Thoracic Surgery, First Medical Center Chinese PLA General Hospital and PLA Medical School Beijing China

**Keywords:** CENP‐F, mechanism, mitosis, tumor

## Abstract

**Background:**

Centromere protein F (CENP‐F) is an important nuclear matrix protein that regulates mitosis and the cell cycle, and plays a crucial role in recruiting spindle checkpoint proteins to maintain the accuracy of chromosome segregation. Studies have shown that CENP‐F is closely involved in the pathogenesis of various diseases, particularly in the development and progression of malignant tumors, where it exhibits significant oncogenic activity.

**Objective:**

This review aims to systematically summarize the molecular structure, subcellular localization, expression regulation, intracellular transport mechanisms, biological functions, and carcinogenic mechanisms of CENP‐F, as well as explore its potential value in cancer diagnosis and therapy.

**Methods:**

A comprehensive review and analysis of domestic and international research literature related to CENP‐F were conducted, focusing on its role in tumorigenesis, development, and as a therapeutic target.

**Results:**

CENP‐F acts as an oncogene and can maintain or promote the malignant phenotype of tumor cells through multiple mechanisms, including regulating signaling pathways related to cell proliferation and apoptosis, promoting metabolic reprogramming, angiogenesis, and tumor cell invasion and metastasis. Additionally, it plays an important role in the immune microenvironment and drug resistance regulation.

**Conclusion:**

CENP‐F plays a key, multidimensional role in tumor biology and is a promising therapeutic target for cancer treatment. Further exploration of the core pathways through which CENP‐F regulates tumorigenesis and its potential for clinical translation is needed.

## 1 Introduction

Cancer is a significant contributor to global mortality, accounting for approximately 9.7 million deaths in 2022 [1]. Cancer arises from genetic mutations that lead to cell abnormalities and uncontrolled growth. The following characteristics are typical of this disease: The hallmarks of cancer include proliferative signaling; evasion of growth inhibition and immune clearance; the ability to replicate indefinitely; tumor proinflammatory action, activation of invasion and metastasis, pro‐angiogenesis, genomic instability and mutation, resistance to cell death, uncontrolled cellular energy metabolism, unlocked plasticity, non‐mutational epigenetic reprogramming, polymorphic microbiome, and senescent cells [2]. However, the occurrence and development of malignant tumors are the result of the combined influence of tumor cells and the tumor microenvironment (TME). Tumor cells release cell signaling molecules into the microenvironment, promoting angiogenesis and inducing immune tolerance; these factors then interact with and regulate tumor cells, forming a large and complex immune microenvironment [3]. The driver genes play a pivotal role in the development and biological effects of tumors. The mutation of driver genes confers selective advantages on tumor cells, while the inactivation of tumor suppressor genes or the activation of proto‐oncogenes results in cell abnormalities [4, 5]. The rapid advances in precision cancer diagnosis and treatment are closely related to progress in cancer driver gene research. Indeed, research on cancer driver genes and their action pathways has identified potential therapeutic methods and drug targets for elucidating the disease mechanism [6].

The centromere plays a pivotal role in the transmission of genetic material to the next generation during mitosis or meiosis in eukaryotes. It ensures the accurate transmission of genetic information from parents to offspring and regulates the proper separation and arrangement of chromosomes as well as spindle assembly during cell division [7]. Abnormal centromere function can lead to chromosomal instability due to chromosome dislocation, resulting in aneuploidy, genome rearrangement, and the formation of micronuclei. Misregulation of the centromere promotes unlimited cell proliferation and tumor development [8]. The centromere is the region of chromosomes where the kinetochore is constructed; it connects the two chromatids in eukaryotic chromosomes and divides them into long arms and short arms [9]. From the outer to the inner layers, a centromere comprises three parts: a dynamic domain, a central domain, and a pairing domain. The kinetochore, a protein disk‐like structure composed of two layers of proteins attached to either side of the centromere, serves as the interface between the chromosome and the spindle. The outer layer of the kinetochore is connected to the spindle filament microtubules, while the inner layer is firmly attached to chromatin within the core region of the centromere [10].

In mammals, the function of the centromere relies on a family of centromere proteins (CENPs) [11], which encompasses both fixed (CENPA, CENPB, CENPC, CENPD, CENPG, CENPH, and CENPI) and transient (CENPE and CENP‐F) structures [11]. CENPA, the pioneering centromere protein identified, plays a pivotal role in the assembly and formation of the centromere, ensuring proper chromosome separation and normal cell division [12]. CENPE, on the other hand, facilitates the regulation of the interaction between the centromere and the spindle; its absence leads to an abnormal arrangement of chromosomes during mitosis, eventually causing mitosis to stop at the M phase [13]. High expression of these CENP family genes affects tumor development by promoting cell cycle progression [14]. Moreover, CENPs regulate the tumor mutational burden and microsatellite instability and are associated with tumor immunity and chemotherapy sensitivity [15]. CENPA expression is also associated with local and metastatic recurrence [16], CENPB and CENPC are related to poor overall survival and disease‐free survival [17, 18], and CENP‐F and CENPE mutate more frequently in various tumors than CENPM and CENPA [15]. Furthermore, CENPH is significantly related to the immune microenvironment and RNA modification and promotes tumor development [19]. In recent years, research has gradually highlighted CENP‐F as a gene that is closely related to malignant tumors. In 1993, Rattner was the first to identify CENP‐F in the serum of human patients with autoimmune diseases [20]. Subsequent studies have shown that CENP‐F is associated with the cell cycle, cell death, embryogenesis, and tumorigenesis [21]. In this study, the authors offer a comprehensive review of CENP‐F from the perspective of its structure, localization, expression, intracellular transport mechanisms, biological functions, and carcinogenic mechanisms.

## 2 CENP‐F Structure

**FIGURE 1:**
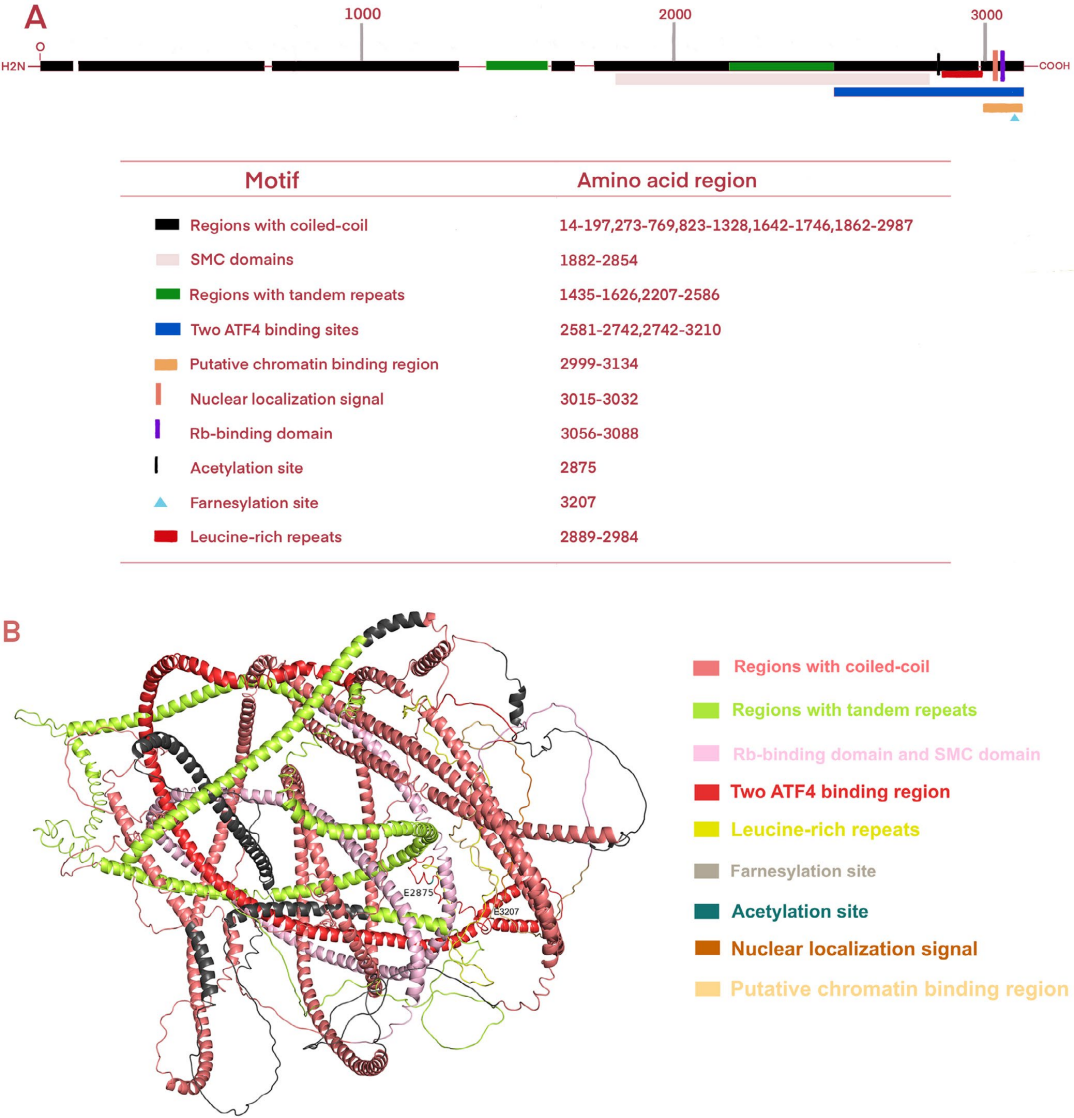
CENP‐F primary structure (A) and Alphafold2 predicted monomer PDB atomic model (B). CENP‐F is a member of the CENPs family of proteins. CENP‐F contains coiled coils, tandem repeats, leucine zips, chromosome structure maintenance domains, and functional molecular binding domains such as chromatin, retinoblastoma protein Rb, and transcription factor ATF4.

Centromere protein F (CENP‐F), a member of the CENP family characterized by the largest molecular weight among mammalian CENPs, has been independently identified as mitosin. CENP‐F is a 367‐kDa nuclear matrix protein comprising 3210 amino acids (Figure 1A) The CENP‐F protein structure predicted by Alphafold2 comprises two winding domains measuring 1600 amino acids in length. Two rings encircle a volatile core from the side, and an ATP‐binding motif with the nucleotide binding site (ADIPTGKT) is present at the spherical carboxyl terminal [22] (Figure 1B) CENP‐F encompasses multiple structural features and functional molecular binding regions, such as coiled helices, tandem repeats [22], the leucine zipper [23], the chromosome structure maintenance domain [23], retinoblastoma protein [24], and the transcription factor ATF4 [25]. In addition, CENP‐F contains a bipartite nuclear localization sequence [24] and post‐translational modifications through phosphorylation [24, 25], acetylation [26], and farnesylation [27] can affect CENP‐F activity and function. Specifically, the phosphorylation site is specific to CDK1, and CDK1 is active in the G2 phase [28]. During the G2/M transition of the cell cycle, the phosphorylation of centromeric protein F (CENP‐F) is critical to the mitosis process. Phosphorylation is a key mechanism that changes protein function. CENP‐F phosphorylation affects its localization and dynamics in the cell, thereby regulating the spatial recombination of centromere and spindle. This recombination is crucial to ensure that chromosomes are precisely allocated between the two daughter cells when the cell divides. Nevertheless, the specific rate at which CENP‐F migrates within the cell and its effect on the mitotic process still need further research to gain a deeper understanding [24]. Acetylation is a key post‐translational modification of proteins that is essential for a variety of cellular processes. For centromeric protein F (CENP‐F), acetylases such as p300/CBP can perform acetylation on its specific lysine residues, affecting the structure, interaction, and stability of CENP‐F. This modification alters the conformation of CENP‐F, thereby affecting its interaction with other proteins or altering its localization within the cell. For example, acetylation can prevent CENP‐F from binding to certain proteins, affecting their function during mitosis. Secondly, acetylation can also regulate the stability of CENP‐F by affecting its ubiquitination and proteasome degradation processes, changing its half‐life in cells, and thus altering the function of CENP‐F. Although the specific acetylation sites and detailed regulatory mechanisms have not been fully elucidated, acetylation clearly plays an important role in the functional regulation of CENP‐F [26, 29]. Lastly, farnesylation is a key protein post‐translational modification process that affects the stability, subcellular localization, and binding efficiency of CENP‐F to other molecules such as microtubules, CENPE, molecular motor proteins, and signal transduction molecules by adding farnesyl groups to specific cysteine residues of proteins such as CENP‐F. However, the factors leading to the farnesylation of CENP‐F and its binding mechanism with other molecules remain unclear, and further studies are needed to prepare targeted inhibitors.

## 3 Localization and Expression of CENP‐F

**FIGURE 2:**
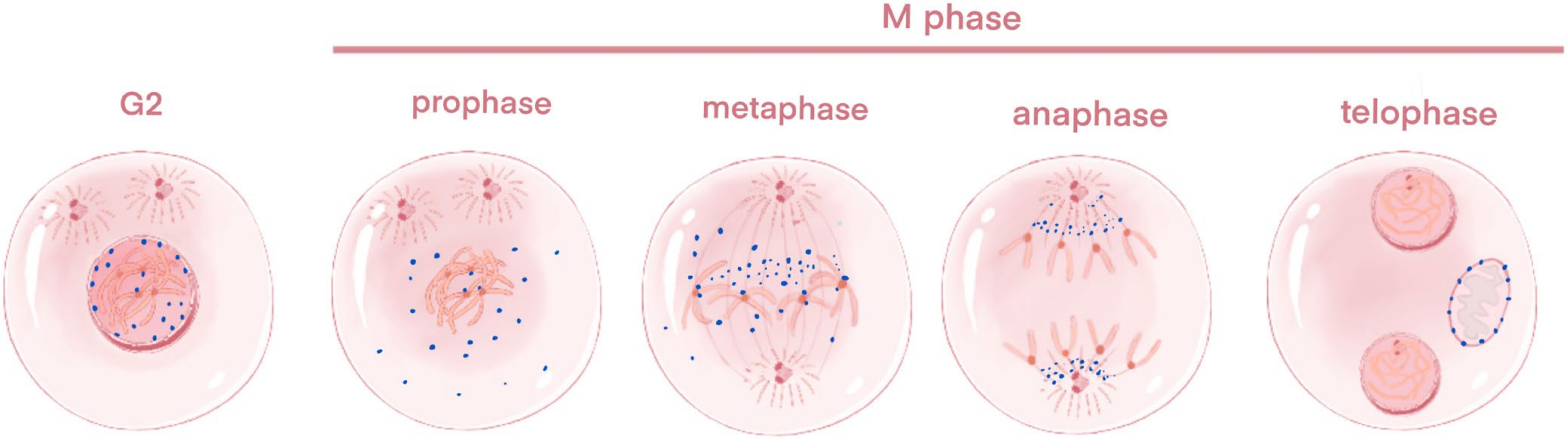
Localization and expression of CENP‐F during mitosis. CENP‐F was located in the nucleolar and nuclear membrane during G2. During the prophase and metaphase stages, it was found in the cytoplasm and centromere. In anaphase, CENP‐F was positioned in the middle of the spindle. Finally, in telophase, CENP‐F degraded after migrating to the mitochondrial outer membrane.

CENP‐F is a cell‐cycle‐associated nuclear antigen localized to the outer surface of the kinetochore region of the centromere and the midpoint of the spindle. Its instantaneous expression and subcellular localization patterns are regulated in a cell cycle‐dependent manner, analogous to the regulation observed for “chromosome passenger proteins”. Specifically, CENP‐F depolymerizes from the kinetochore upon the completion of cell division [30]. During the G0/G1 phase, the expression level of CENP‐F is low in cells. Conversely, in the S phase, CENP‐F accumulates in the nuclear matrix, with minimal expression in the centrosomes and mitochondria, achieving maximum expression in G2/M cells [31]. As the cell transitions into the early G2 phase, the protein becomes soluble within the nucleus and is present in granular form within the non‐nucleolar region. Subsequently a portion of the protein accumulates on the nuclear membrane, while the majority remains soluble within the nucleus [22]. At the conclusion of the G2 phase, CENP‐F accumulates at the centromere earlier than any other known transient CENP. During the M phase, CENP‐F exhibits a dynamic spatial and temporal distribution across the nuclear membrane. The main difference between CENP‐F and other types of CENP proteins, such as CENPA, exhibit cell cycle‐dependent expression and rapid degradation, whereas CENPA, as A variant of the centromere's histone H3, is relatively stable during the cell cycle [22]. As the nuclear envelope undergoes breakdown and subsequently disappears, CENP‐F within the nucleus diffuses into the cytoplasm, with a portion of CENP‐F remaining anchored to the outer centromere region until the transition from metaphase to anaphase [20]. During the anaphase stage of mitosis, CENP‐F initially localizes in the middle of the spindle, and subsequently recruits the spindle checkpoint regulatory complex to the spindle pole, facilitated by the migration of cytoplasmic dynein‐1. At the telophase stage of mitosis and during the early G1 phase, CENP‐F is recruited by Miro into the mitochondrial network and participates in its normal distribution; mitochondrial network is a network structure formed by the interconnection of mitochondria in the cell, which is crucial for mitochondria to perform their functions efficiently, such as producing energy (ATP), participating in cell metabolism, regulating cell death, etc. Miro, as a calcium‐binding GTPase on the outer membrane of mitochondria, through direct interaction with CENP‐F, promotes the transport and distribution of mitochondria to the cell periphery, which is very important for the reconstruction and maintenance of the normal function of the mitochondrial network in the daughter cells after cell division, and cells lacking CENP‐F or Miro will show reduced mitochondrial network diffusion and transport defects, thus confirming the role of Miro and CENP‐F in the normal distribution of mitochondria [32] (Figure 2).

CENP‐F expression is regulated by a variety of epigenetic modifications, such as methylation and acetylation, and copy number variants, while not directly regulating its expression, have important effects on its function. Shi et al. utilized cBioPortal to analyze the genetic changes of CENP‐F (amplification, depth deletion, mis‐sense mutation, splicing mutation, and truncation mutation) across different cancers. They discovered that the mutation frequency of the CENP‐F gene was 12%, with a single nucleotide polymorphism mutation frequency of 48%. Compared to CENP‐F, the CENPE gene mutated 3% of the time in tumors, while CENPM and CENPA mutated even less frequently at 3% and 2.4%, respectively. This suggests a relatively high frequency of CENP‐F mutations in CENP family genes, which may be related to its specific role in tumorigenesis. Moreover, the expression level of CENP‐F was directly proportional to its copy number variation, while the methylation level of CENP‐F exhibited a negative correlation with its mRNA expression [15]. These findings suggest a high degree of genetic heterogeneity in CENP‐F. Additionally, activation of histone acetylation leads to failure of CENP‐F to attach to the centromere, thus preventing the mitotic process [29]. The transcription factors SP1, ATF4, E2F1, ATF6α, and FOXM1 also induce CENP‐F expression [33, 34, 35, 36, 37]. Some long non‐coding RNA, such as smooth muscle‐induced long non‐coding RNA and LINC00536, increase the expression of CENP‐F mRNA [38, 39]. At the telophase stage of mitosis, CENP‐F exists on the daughter cell intracellular bridge and is rapidly degraded by farnesylation and ubiquitination at the end of mitosis [31]. CENP‐F is recognized and ubiquitinated by the APC‐Cdh1 ubiquitin ligase via the ubiquitin‐proteasome pathway and subsequently degraded by the proteasome [31, 40]. Farnesylation of CENP‐F does not affect localization at the centromere but is required for degradation through G2/M processes and after mitosis [31, 41].

## 4 Intracellular Transport of CENP‐F

**FIGURE 3:**
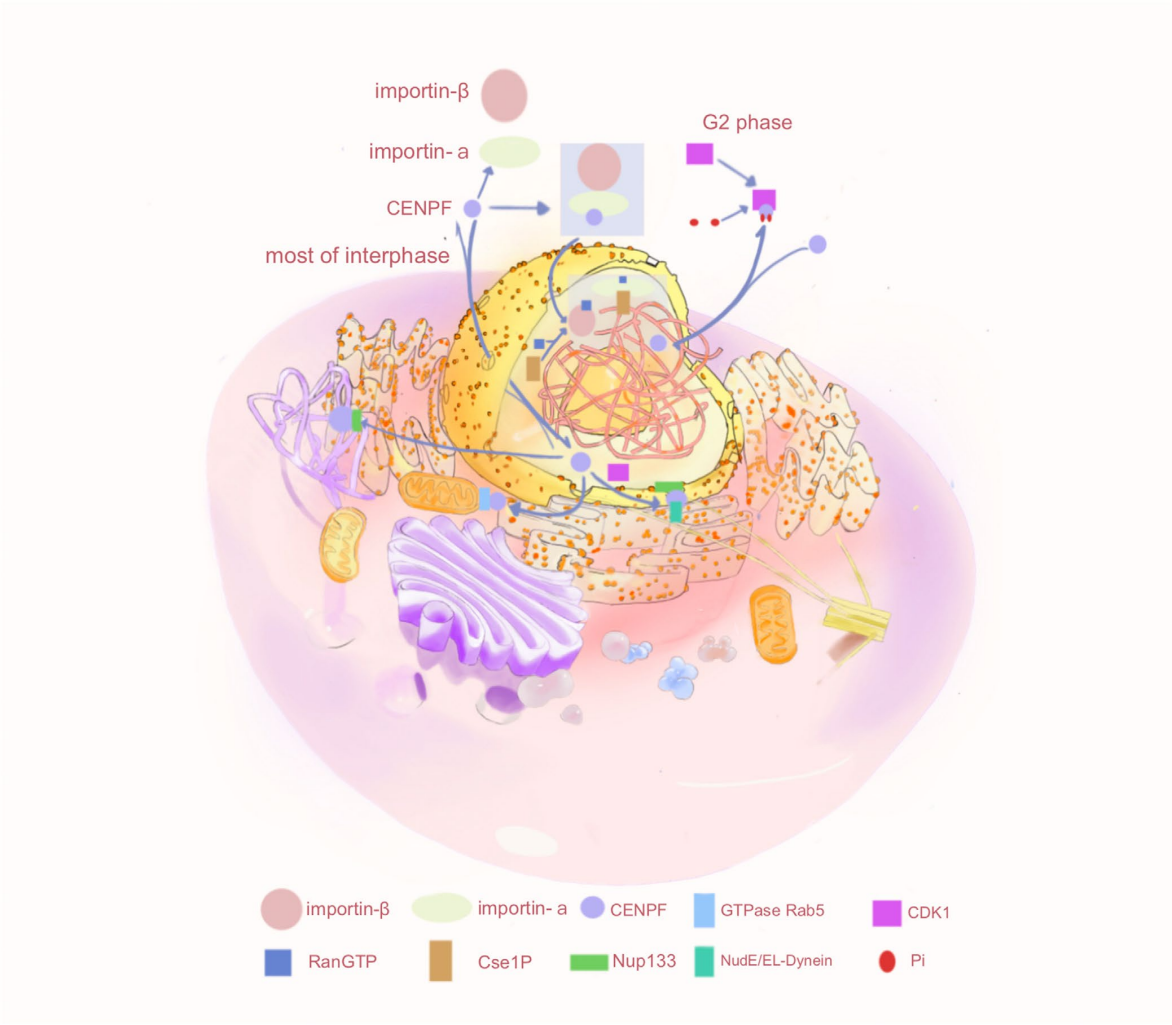
Intracellular transport mechanism of CENP‐F controls its dynamic localization in cells through binding with various proteins, thereby regulating the cell cycle process and thus affecting the occurrence of diseases. It plays a crucial role in the occurrence of diseases through different transport rates in the nucleus–cytoplasm and then binding to the chromosomal and mitochondrial regions.

CENP‐F is a regulator of cell cycle progression, recruiting motor proteins and coordinating cell cycle‐specific transport events, including transport in the nuclear, chromosomal, and mitochondrial regions. CENP‐F transport not only regulates cell division, proliferation, and apoptosis, but also its related functions, such as normal cell division and the occurrence of diseases, are inseparable from its transport (Figure 3).

### 4.1 Transport Mechanisms in the Nuclear Region

During the cell cycle, the subcellular localization of CENP‐F changes. Throughout the majority of the interphase, CENP‐F resides within the nuclear matrix, but it is exported to the cytoplasm during the G2 phase. Prior to the G2 phase, the mechanism responsible for the nuclear import of CENP‐F remained elusive, and Crm was the most frequently implicated nuclear export factor. However, the involvement of Crm in the transport of CENP‐F has yet to be conclusively demonstrated. CENP‐F possesses a classic protein C bipartite nuclear localization signal (cNLS) recognized by the nuclear transport factors importin‐α and importin‐β [42]. After the cNLS binds to nuclear importin‐α, ternary complexes are formed with the nuclear importin‐β and CENP‐F, which are selectively imported from the cytoplasm through the nuclear pore complex in a RanGTP‐dependent manner [43]. CENP‐F is also a shuttle protein that can be actively exported from the nucleus. Phosphorylation of the cNLS of CENP‐F may reduce its affinity for nuclear importin‐α, thereby weakening the cNLS and slowing the rate of nuclear import in the G2 phase, resulting in CENP‐F output into the cytoplasm [28]. CENP‐F then becomes involved in cell cycle processes, centromere assembly, and faithful chromosome separation [28]. However, the functional role leading to specific phosphorylation of cNLS in CENP‐F sequences is still unclear. Therefore, exploring the key factors of the cell signal transduction network after CENP‐F phosphorylation can reveal the network structure and dynamic changes of phosphorylation regulation and provide new targets for early diagnosis and treatment of diseases, as well as directions for drug development. After nuclear export at the end of the G2 phase, the nuclear porin Nup133 recruits CENP‐F to the nuclear membrane, which in turn recruits kinetochore proteins via NudE and NudEL [28]. These proteins directly bind to cytoplasmic dynamin and its regulatory factor LIS1 [44, 45, 46]. The nucleus is positioned relative to the centrosome along the microtubule locus in a kinetochore protein‐dependent manner.

### 4.2 Transport Mechanisms in Chromosomal Regions

CENP‐F is a non‐motile microtubule‐binding protein involved in centromere–microtubule binding [47]. CENP‐F is released from the nuclear membrane via a small GTPase Rab5‐dependent mechanism in the early G2/M phase and subsequently targets the outer plate until it moves to the mature centromere in the later phase [28]. In addition, CENP‐F co‐locates with Nup133 to assist in the recruitment of the Nup107‐160 complex to the centromeres after the disintegration of the nuclear pore complex. CENP‐F may act as an adaptor protein to promote the localization of the Nup107‐160 complex on the centromeres and may further assist in the recruitment of kinetochore proteins. Kinetochore proteins are a class of motor proteins that rely on microtubules and are responsible for transporting goods within cells, including helping chromosomes separate during mitosis [48, 49]. However, when intracellular Ca^2+^ is depleted, the loss of this signaling molecule may affect the stable localization and function of CENP‐F at the centroid through downstream effector molecules such as calmodulin or other calcium ionbinding proteins, resulting in impaired connections between the centroid fibers and the centroid, indirectly disrupting the stability of microtubules and thus blocking the cell cycle process [50]. At present, the other mechanisms leading to CENP‐F detachment from the chromosome centromere are still unclear. Studying the mechanism of CENP‐F detachment can not only improve the transport mechanism of CENP‐F but also provide new drug targets for the treatment of diseases.

### 4.3 Transport Mechanisms in the Mitochondrial Region

CENP‐F is also implicated in the mitotic redistribution of the mitochondrial network [47]. The mitochondrial transport facilitated by CENP‐F is linked to the growth of microtubule tips [47]. CENP‐F is recruited into the mitochondrial outer membrane by the mitochondrial outer membrane protein GTPase Miro during cytoplasmic division, and the Miro–CENP‐F interaction connects the mitochondria to the growing microtubule tip, causing the mitochondria to track along the microtubule tip and be transported to the daughter cell edge at the end of mitosis [32]. In the anaphase stage, CENP‐F is degraded by the late promoting complex. Cells lacking CENP‐F or Miro exhibit reduced expansion of the mitochondrial network and specific defects in the cytoplasmic division of mitochondrial transport to the periphery of the cell, thus affecting normal cell division and leading to the occurrence of disease [51, 52, 53]. However, the pathophysiological and physiological functions of mitochondria caused by CENP‐F are still unclear. Consequently, it is crucial to investigate the morphological and tissue heterogeneity, as well as the pathophysiological functions of mitochondria affected by CENP‐F for the pathogenesis of diseases, and provide a new way for disease treatment. Thus, CENP‐F plays an important role in maintaining cell cycle progression and the occurrence of disease through intracellular transport mechanisms. As such, researchers must delve into the role of CENP‐F in cancer and its carcinogenesis mechanism.

## 5 Biological Function of CENP‐F

CENP‐F is widely involved in the correct assembly of the centromere–microtubule–spindle in cells, as well as the transport of life activities between mitochondria and centrosomes; therefore, its loss or abnormal function can lead to chromosome defects, embryo development retardation, and the formation of disease [54, 55, 56].

### 5.1 CENP‐F Deletion Causes Chromosomal Defects

#### 5.1.1 CENP‐F Deletion Causes Chromosomal Arrangement Defects and Premature de‐Coagulation

To explore the function of CENP‐F, Laoukili et al. silenced CENP‐F with siRNA in U2OS cells; depletion of CENP‐F resulted in mitosis even after intracellular chromosome arrangement defects. Notably, even the introduction of mitotic inhibitors such as nocodazole and taxol failed to arrest the mitosis of these CENP‐F‐depleted cells. The observation that two checkpoint proteins (BubR1 and Mad1) on the centromeres of some CENP‐F‐depleted cells were reduced led the authors to hypothesize that CENP‐F is necessary for sustained activation of the spindle assembly checkpoint (SAC) [57]. In another study, Yang et al. found no inactivation of the SAC when CENP‐F expression was inhibited by siRNA [58], which contrasts with the findings reported by Laoukili et al. [57]. Furthermore, the deletion of CENP‐F resulted in chromosome misarrangement, premature de‐coagulation, and mass cell death. The expression levels of the cell cycle‐related proteins CENPE, cytoplasmic dynamin, and dynamin activating protein were all decreased after CENP‐F deletion, whereas BubR1, Mad2, Bub1, hNuf2, Aurora B, and survivin expression were not affected. Therefore, the authors speculated that the chromosomal orientation and spindle morphology defects in CENP‐F‐depleted cells may be attributed to the decreased stability of CENPE [58]. Additionally, the CAAX domain is a key C‐terminal sequence of some proteins, which enables proteins to participate in important biological processes such as cell signaling, cell cycle control, and protein localization through farnesylation, a post‐translational modification. This process usually involves the addition of a farnesyl group to a cysteine residue and is critical for the localization and function of the protein. Farnesyl transferase inhibitors (FTIs) cause defects in chromosome alignment during mitosis by blocking farnesylation of the C‐terminal CAAX domain of CENP‐F. This inhibitory effect may lead to tumor cell cycle arrest and inhibit tumor growth, showing the potential of FTIs as an anticancer therapeutic strategy [59].

#### 5.1.2 CENP‐F Deletion Causes Chromosome Mis‐Segregation and Delayed Mitosis

Holt et al. found that the loss of CENP‐F leads to premature chromosome separation, a failure in cytoplasmic division, and a delay in mitosis [60]. This delay may be attributed to chromosome misalignment caused by the deletion of CENP‐F. The co‐deletion of the checkpoint kinase BubR1 and CENP‐F prevents mitotic arrest, suggesting that SAC is activated by CENP‐F deletion [60]. This is in contrast with the conclusion of Laoukili that the loss of CENP‐F causes SAC inactivation [57]. According to Bomont et al., the absence of CENP‐F induces two distinct phenotypes, potentially depending on the number of CENP‐F molecules present at the centromere [61]. This discovery further enriches our understanding of the function of CENP‐F. In one of the phenotypes, CENP‐F knockdown occurs in all cells, resulting in serious chromosome mis‐segregation and failure of centromere assembly. In the other phenotype, cells with partial CENP‐F knockdown show delayed mitosis, decreased centromere–microtubule stability, and reduced intercentromere tension of bidirectional metaphase chromosomes [61]. Hussein and Taylor investigated the mechanism of action of farnication‐dependent CENP‐F in regulating chromosome separation and SAC signaling and found that cell division was delayed when 630 amino acids (C630) were present at the C‐terminal of CENP‐F, but chromosome separation and mid‐late transition were not inhibited [27].

### 5.2 Deletion of CENP‐F Retards Fetal Development

Zhou et al. studied the function of CENP‐F in the early embryogenesis of mice, using the Morpholino and Trim‐away methods to specifically interfere with CENP‐F expression, and found that the loss of CENP‐F function directly prevented pre‐implantation embryo development [54]. These results provide a new understanding of the mechanism of chromosome separation regulation and heteroploidy‐related diseases in the process of embryonic cell division.

### 5.3 CENP‐F Deletion or Mutation Causes Disease

CENP‐F deletion or mutation can also lead to the development of various diseases. CENP‐F, a microtubule regulatory protein, serves a dual role as a CENP in spindle orientation and cilia development [62, 63]. In addition, CENP‐F emerges as a novel centripetal disease gene, where mis‐sense mutations and truncations within it, particularly in asymmetrically divided stem cells, are associated with severe human ciliosis and microcephaly‐associated phenotypes [62]. Biallelic loss‐of‐function variation in the CENP‐F gene leads to Strømme syndrome (a disease characterized by intestinal atresia, anterior chamber abnormality, and microcephaly). Furthermore, CENP‐F knockout mice have revealed structural kidney defects, encompassing the loss of ciliary structure, renal tubule dilatation, and glomerular destruction, which suggests that CENP‐F proteins play a role in kidney development [56]. Filges et al. also observed that CENP‐F mutations can lead to intestinal atresia in humans [64, 65]. The details of how mutations or deletions of CENP‐F cause disease are still unclear. Thus, the loss or mutation of CENP‐F can cause different types of damage to various organs, highlighting the need to further explore the function and mechanism of CENP‐F.

## 6 Role and Regulatory Mechanism of CENP‐F in Cancer

### 6.1 Role of CENP‐F in Cancer

CENP‐F is a carcinogenic protein implicated in the development of numerous cancer types, with its elevated expression linked to malignant tumor progression and unfavorable clinical outcomes. In hepatocellular carcinoma cells, high CENP‐F expression is positively correlated with the serum marker AFP, venous invasion, advanced differentiation stages, and shorter overall survival. Furthermore, it promotes cell proliferation, migration, and invasion and contributes to the accumulation of tumor mutational burden and microsatellite instability [66, 67]. CENP‐F and FOXM1 serve as crucial regulators of prostate cancer malignancy and are indicative of poor tumor survival and extensive metastasis [68]. Immunohistochemical analysis in nasopharyngeal carcinoma showed that CENP‐F expression was positively correlated with clinicopathological features and negatively correlated with overall survival. Notably, CENP‐F expression is an independent predictor of cancer prognosis [69]. For instance, high CENP‐F expression negatively correlates with the prognosis of triple‐negative breast cancer and lung adenocarcinoma [33, 70], and CENP‐F has been highlighted as a potential prognostic biomarker in liposarcoma [71]. Additionally, CENP‐F expression is significantly elevated in the supernatant of liver tumor cells. Overexpression and abnormal localization of the CENP‐F autoantigen can trigger the production of CENP‐F autoantibodies, which are considered potential serum biomarkers for early liver cancer diagnosis [30].

CENP‐F can not only promote the malignant progression of tumors but may also have some guiding significance for the treatment of tumors. For instance, CENP‐F enhances doxorubicin resistance in triple‐negative breast cancer by modulating the CHK1‐mediated G2/M phase arrest. Silencing CENP‐F can sensitize these cancer cells to both radiation and chemotherapy [33]. In the context of liposarcoma, high CENP‐F expression is associated with a poor immune score and emerges as a potential indicator of malignant outcome related to immunoinfiltration‐based survival [71], which suggests that liposarcoma may not benefit from immunotherapy. FTIs, which represent one of the earliest attempts to target the RAS oncogene for cancer therapy, can also target CENP‐F through its deactivation [72]. Some studies have shown that zoledronic acid can inhibit the farnesylation of CENP‐F, which occurs in the S phase and pre‐division phase, and destroy the normal localization and function of CENP‐F [73]. FTIs have also shown strong cytotoxicity as a single drug in clinical studies and promising clinical applications in combination with other therapeutic strategies [74]. Therefore, targeted CENP‐F treatment combined with immunotherapy may be an attractive therapeutic strategy for cancer. However, such attempts require further elucidation of the carcinogenic mechanism of CENP‐F.

### 6.2 Regulatory Mechanisms of CENP‐F in Cancer

**FIGURE 4:**
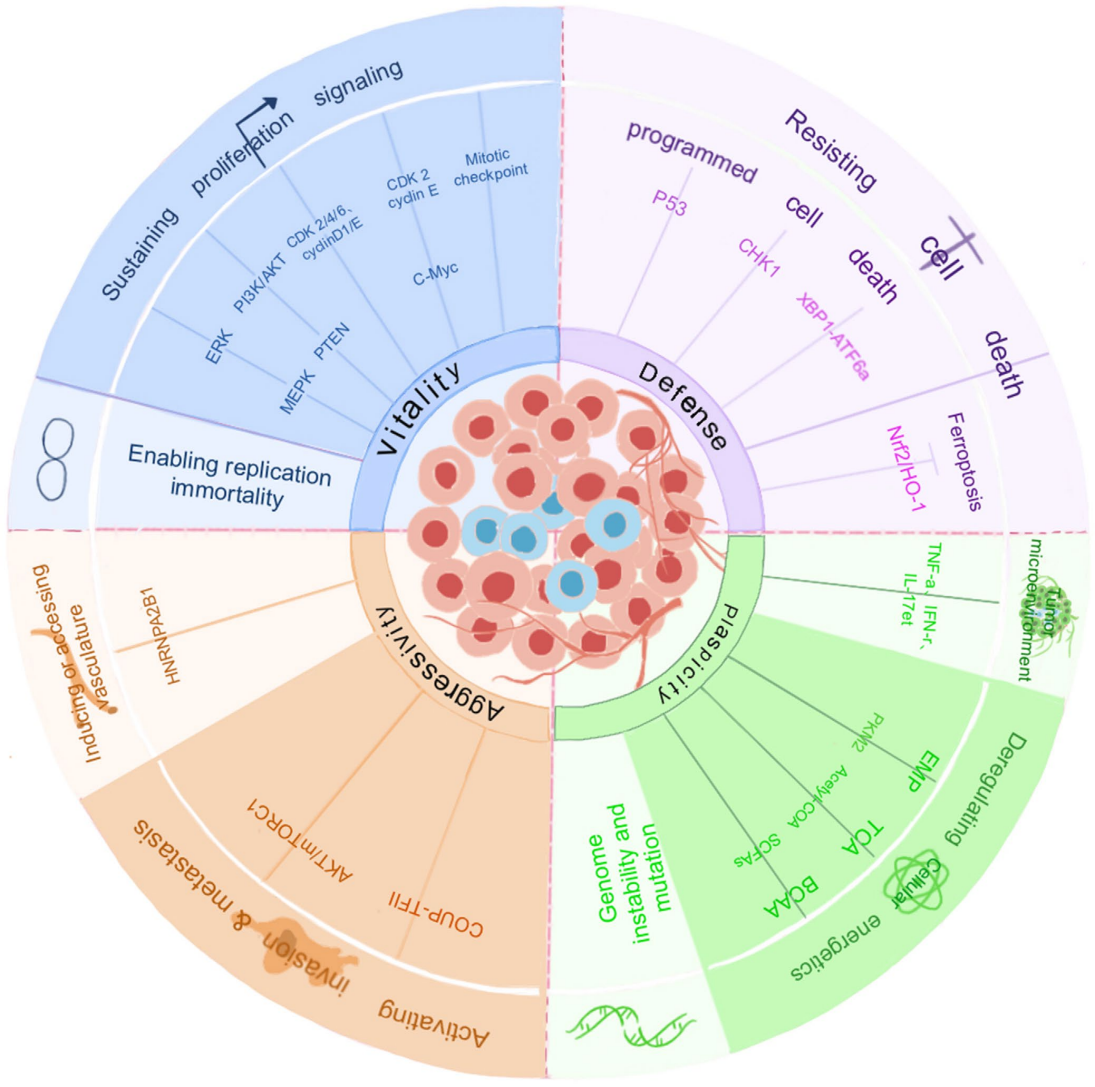
Carcinogenic characteristics of CENP‐F. CENP‐F mainly promotes the occurrence and development of malignant tumors through four aspects: Vitality (sustained cell proliferation signal and enabling replication immortality), defense (resisting cell death), aggressivity (inducing accessing vasculature and activating invasion and metastasis) and plasticity (genomic instability and mutation, deregulating cellular metabolism and TME).

**FIGURE 5:**
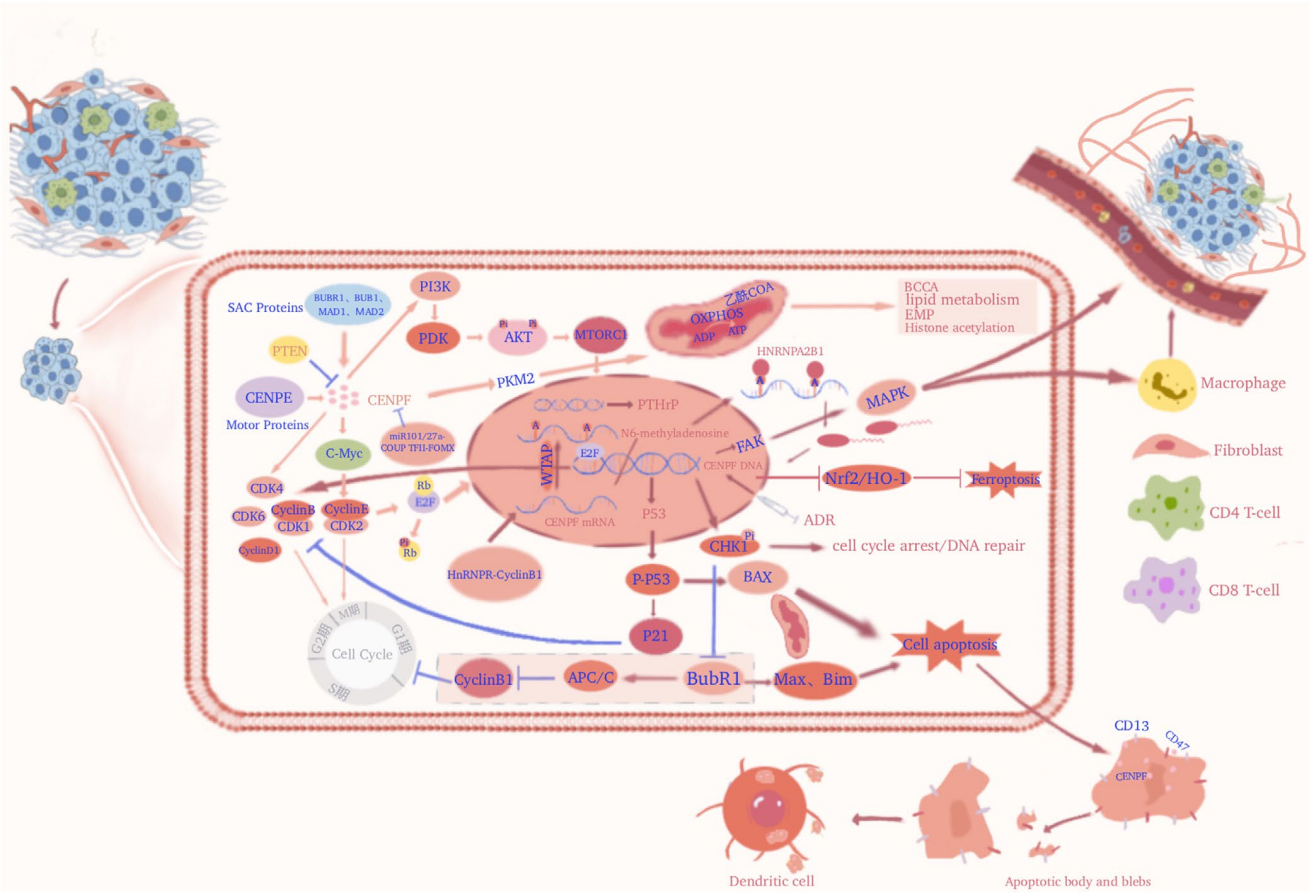
Mechanism of CENP‐F in cancer. The regulatory role of CENP‐F in cancer mainly depends on the signaling pathways responsible for cell proliferation, resistance to apoptotic cell death, metabolic reprogramming, activation of invasion and metastasis, induction of blood vessel formation, protein pharnylation, and promotion of tumor development, invasion, and metastasis in the tumor microenvironment.

The exact mechanism by which CENP‐F regulates the onset of cancer remains an open question. However, exploring cell proliferation or apoptosis, metabolic reprogramming, invasion, metastasis, and the TME can provide insights into its regulatory role in cancer (Figures 4 and 5).

#### 6.2.1 Control of Proliferating Signals

Cell division is driven by intracellular signals, typically initiated by contact between growth factors and congener receptors, which ultimately activate transcription factors and express the proteins required for cell cycle progression. CENP‐F regulates the cell cycle by recruiting factors associated with proliferation signaling and is therefore critical for tumor development.

First, CENP‐F determines cell proliferation by regulating the mitogen‐activated protein kinase (MAPK)/extracellular signal‐regulated kinase signaling pathway, and more precise mechanisms need to be studied [75]. CENP‐F knockdown may inhibit the progression of lung adenocarcinoma by inhibiting the estrogen receptor β 2/5 pathway [76].

Second, CENP‐F overexpression is clearly associated with PTEN deletion, indicating a functional relationship between the two genes. Deletion of PTEN is the main cause of the highly active PI3K/AKT signaling pathway in prostate cancer and is associated with tumorigenesis and adverse clinical outcomes [77]. A functional interaction between CENP‐F and the PI3K/AKT signaling pathway has been observed in DU145 prostate cancer cells, where the PI3K/AKT signaling pathway is completely eliminated when CENP‐F and FOXM1 are co‐silenced [78].

Third, CENP‐F can inhibit mitosis by directly targeting cyclins. CENP‐F knockdown inhibits the proliferation of hepatocellular carcinoma cells by inhibiting the expression of cyclinD1, c‐Myc, CDK2, and CDK4, but has no effect on apoptosis or necrosis [30]. CENP‐F expression is related to the overexpression of cyclin E, which activates the cyclin E/CDK2 complex and leads to the acceleration of the S phase [73]. Overexpressed CENP‐F up‐regulates CDK1‐mediated G2/M phase transformation [67]. Furthermore, CENP‐F knockdown can significantly reduce the proliferation of renal cell carcinoma and regulate the cell cycle by inhibiting the expression of CDK4, CDK6, cyclinD1, and other cyclins [79].

Fourth, CENP‐F expression is also associated with c‐Myc amplification, which can activate cyclin E/CDK2, leading to cell cycle progression and proliferation [80]. In addition, CENP‐F expression significantly contributes to high telomerase activity. Telomerase activation is associated with telomere dysfunction, which is the primary mechanism of chromosomal instability in human cancer [81, 82]. Furthermore, a notable proportion of tumors that overexpress CENP‐F exhibit aneuploidy, thus affirming the connection between CENP‐F expression and markers of chromosomal instability [73].

Fifth, CENP‐F can bind to mitotic checkpoint proteins to regulate cell proliferation. But in 5.1, it is described that the involvement of CENP‐F in SAC is controversial. In human cells, Bub1 is dependent on Bub3 and initially localizes in the inner and outer centromere plates for recruitment, after which CENP‐F accumulates at the centromere [83, 84]. This is followed by the centromere accumulation of BubR1, CENPE, and Mad2 [85, 86]. The centromere motor proteins CENPE and CENP‐F are essential for the correct attachment of microtubules and the regulation of spindle checkpoints [87, 88]. Indeed, centromeres devoid of CENP‐F show reduced amounts of the mitotic checkpoint proteins BUBR1, Mad1, Mad2, hBUBR1, hBUB1, and hMps1, which slow cell‐cycle progression [89].

#### 6.2.2 Resistance to Cell Death

The cell death pathway mainly includes apoptosis, autophagy, necrosis, programmed necrosis, and iron death. Apoptosis and iron death play an important role in ontological development and the development of various diseases, and resistance to apoptosis is a hallmark of cancer cells. Iron death is a mode of cell death associated with iron metabolism disorders characterized by increased intracellular active iron content and production of reactive oxygen species (ROS), leading to lipid peroxidation of the cell membrane and cell death. Endogenous pathways usually refer to cell death processes triggered by factors inside the cell, such as mitochondrial dysfunction or DNA damage. Recent studies have shown that CENP‐F is involved in the regulation of apoptosis mechanisms and iron death pathways [67, 90, 91]. CENP‐F regulates apoptosis mainly through endogenous pathways and plays an important role in the mitochondrial pathway, DNA damage, and endoplasmic reticulum stress response [92].

CENP‐F plays a regulatory role in the level of P53 in cells, which is a well‐characterized tumor suppressor. Specifically, in adrenal cortical carcinoma, overexpression of CENP‐F activates p53‐mediated anti‐tumor effects by inducing P‐p53, P21‐mediated G2/M phase cell cycle arrest, or BAX‐mediated apoptosis [67]. CENP‐F also regulates CHK1‐mediated cell cycle arrest in the G2/M phase. CHK1 is a key protein in the G2/M phase checkpoint that is involved in the DNA damage response caused by endogenous and exogenous factors and is critical for genome stability and cell survival [93]. Once DNA repair is disrupted, the main process shifts from cell cycle arrest to apoptosis [33]. In ER−/PR−/HER2− breast cancer, ADR doxorubicin activates CHK1, regulates cell cycle arrest mediated by the mitotic checkpoint complex–APC/C–cyclinB1 axis, and regulates apoptosis induced by MSX2 and BIM (bcl‐2) [90].

However, endoplasmic reticulum stress plays a crucial role in CENP‐F expression. Under endoplasmic reticulum stress, CENP‐F overexpression inhibits apoptosis and promotes cell proliferation [36]. Endoplasmic reticulum stress also induces XBP1 to bind to ATF6α, resulting in increased DNA binding affinity, which inhibits the transcriptional activity of the CENP‐F gene and inhibits cell proliferation [36].

In the iron death pathway, CENP‐F knockdown increases the expression levels of ROS and lipid peroxidation products (MDA) and induces the iron death of cervical cancer cells by triggering the Nrf2/HO‐1 signaling pathway, thereby inhibiting cell proliferation, migration, and invasion [91].

#### 6.2.3 Regulation of Metabolic Reprogramming

The “Warburg effect” in tumorigenesis is one of the most widely studied models of cellular metabolic reprogramming, whereby cancer cells autonomously alter the flow of various metabolic pathways to meet increased biological energy and biosynthesis requirements and reduce the oxidative stress required for cancer cell proliferation and survival [94].

CENP‐F may regulate the metabolism of cancer cells by regulating the phosphorylation of pyruvate kinase M2 (PKM2). For example, Shahid et al. used the CRISPR‐Cas9 system to silence the CENP‐F gene in human PC3 cells, which resulted in a general decrease in metabolic activity in PC3 cells [95]. CENP‐F deletion increases inactive PKM2, and phosphorylation of active PKM2Tyr105 impaired the enzyme activity of PKM2, which reduced the ability of the phospho‐group of 2‐phosphoenolpyruvate to catalyze ADP to form ATP, reduced the production of pyruvate, reversed the epithelial–mesenchymal transition, and inhibited the metabolic rate by reducing oxidative phosphorylation and glycolysis [95, 96, 97, 98, 99]. Additionally, CENP‐F deletion reduces the overall biological energy capacity, acetyl‐CoA production, histone acetylation, and lipid metabolism, suggesting that CENP‐F is a key regulator of cancer metabolism [95]. Furthermore, CENP‐F can also control prostate cancer invasion and progression through branched‐chain amino acid metabolism. Acetate, one of the main short‐chain fatty acids, is a key substrate for cancer bioenergetics [100]. The synthesis of fatty acids requires acetic acid and acyl‐CoA synthetase short‐chain family member 2. CENP‐F regulates branched‐chain amino acid catabolism depending on the change in acetic acid concentration, thereby controlling tumor invasion and progression [99].

#### 6.2.4 Promotion of Angiogenesis and Activation of Invasion and Metastasis

CENP‐F expression is up‐regulated in gastric cancer and promotes the metastasis of gastric cancer both in vivo and in vitro. Mechanically, the m6A modification of CENP‐F is recognized by heteroribonucleoprotein A2/B1 to promote the stability of its mRNA; increased m6A modification of CENP‐F promotes metastasis and angiogenesis of gastric cancer through CENP‐F/FAK/MAPK and epithelial–mesenchymal transformation axes [101]. In breast cancer cells, high expression of CENP‐F activates AKT phosphorylation and mTORC1 and regulates PTHrP, which modifies the bone microenvironment and promotes the metastasis of breast cancer cells [102]. Furthermore, the COUP–TFII–FOXM1–CENP‐F axis regulated by miR‐101 and miR‐27a promotes the metastasis of prostate cancer [35].

#### 6.2.5 Mechanism of CENP‐F in the Tumor Microenvironment

The TME involves immune cells, stromal cells, blood vessels, and the extracellular matrix, which is important for cancer progression, including tumor development, transfer, and growth. During the apoptosis of hepatocellular carcinoma cells, CENP‐F is transferred into apoptotic vesicles, where it relocates to the outer surface of the plasma membrane [30]. Apoptotic cell components, including intact cells, apoptotic bodies, and apoptotic vesicles, are immunogenic and can induce antibody production [103]. Apoptotic bodies and vesicles exhibit lower expression of CD31 and CD47 and induce dendritic cell phagocytosis and maturation more effectively than intact apoptotic cells, thus activating the human immune response to promote the production of CENP‐F antibodies; this dendritic cell response is independent of CENP‐F expression [30].

CENP‐F exhibits a positive correlation with the gene expression levels of interferon‐gamma, tumor necrosis factor‐alpha, and interleukin‐17 in peripheral blood [104]. In addition, CENP‐F expression is positively correlated with the percentage of CD8^pos^ T cells and negatively correlated with the percentage of CD4^pos^ T cells in peripheral blood. In cutaneous melanoma, CENP‐F is associated with CD4^+^ T cell markers; high CENP‐F expression is negatively correlated with CD4^+^ T cell survival regulators, leading to premature CD4^+^ T cell depletion and immunosuppression [105]. In lung adenocarcinoma, a significantly higher infiltration of CD4^+^ Th2 cells is observed in the CENP‐F‐positive group [106]. Within the microenvironment of liver cancer cells, CENP‐F also positively correlates with inhibitory checkpoint molecules such as PD‐1, CTLA‐4, and TIM‐3, suggesting its involvement in the weakening of anti‐tumor immunity in liver cancer cells [104]. To evaluate the relationship between CENP‐F and the liposarcoma TME, Chen et al. conducted a correlation analysis between the immune cell score and CENP‐F expression; some immune cells in the liposarcoma TME may be related to CENP‐F expression. In particular, the immune scores of “CD4^+^ T cells,” “macrophages”, “M1 macrophages”, and “monocytes” are significantly negatively correlated with CENP‐F expression [71]. This suggests that CENP‐F promotes tumor development in the TME.

By studying the mechanism of action of prostate cancer and cancer‐associated fibroblasts, Zhai et al. identified CENP‐F as a prognostic biomarker of cancer‐associated fibroblasts through single‐cell RNA sequence analysis; moreover, gene set variation analysis showed significant differences between low and high expression of immune checkpoint‐related genes (CD274, CTLA4, HAVCR2, LAG3, PDCD1LG2, TIGIT, and SIGLEC15) in the CENP‐F group [107]. Therefore, changes in CENP‐F expression may be a result of changes in the immune microenvironment. As such, exploring the exact mechanism of CENP‐F in the TME has implications for effectively preventing the occurrence, development, invasion, and metastasis of tumors by providing cancer treatment strategies that target the TME.

## 7 Role of CENP‐F in Tumor Therapy

Elucidating the molecular mechanisms underlying tumor biology and discovering novel, specific biomarkers with diagnostic significance is crucial for providing precise, targeted therapies and enhancing patient survival rates and prognosis. Yong and colleagues, for instance, leveraged bioinformatics to analyze potential therapeutic targets and small molecule drugs for early‐stage lung adenocarcinoma. They identified CENP‐F as a promising biomarker for this condition and Aminopurvalanol A, a small molecule drug, as a potential therapeutic target [108]. In another study, bioinformatics was used to identify CENP‐F as a pivotal target for esophageal squamous cell carcinoma and Amentoflavone as a potential cell cycle inhibitor targeting cyclinB1 [109]. Current evidence indicates that autoimmunity is associated with the malignant incidence and treatment of tumors. For example, in a multicenter prospective study, serum samples were collected from patients with rheumatoid arthritis in an attempt to predict the clinical response of new types of serum autoantibody (ANA) to tumor necrosis factor inhibitors. For the first time, the researchers reported different clinical responses of the new ANAs to different inhibitors. IgG antibody levels of CENP‐F were significantly higher in patients who responded to infliximab (*N* = 44) than in those who did not respond to infliximab (*N* = 111) (*p* < 0.05). Therefore, high expression of CENP‐F IgG antibodies in rheumatoid arthritis is related to the efficacy of infliximab treatment and can help guide treatment strategies [110]. Furthermore, a study examining ANAs in 347 patients with non‐Hodgkin lymphoma emphasized cell cycle‐associated proteins, particularly CENP‐F, as one of its targets [111]. In addition, a multicenter retrospective study reviewing the prevalence and clinical relevance of rare ANAs by continuously testing 1235 serum samples with indirect immunofluorescence assay found that the prevalence of rare ANAs was higher than expected. Therefore, the clinical application of ANAs provides powerful diagnostic information for autoimmune diseases [112]. At present, new anti‐mitotic inhibitors targeting anti‐tumor therapy have only reached the clinical trial stage; however, toxicity and drug resistance are still the most serious problems. Microtubule‐targeting drugs such as vinca alkaloids, taxanes, and ebomycin have shown good clinical efficacy. Moreover, most novel mitotic specific targets have remained in phase I/II clinical trials, such as CDK1, PLK1, CENPE, the NDC80‐NEK2 complex, and Aurorakinase A/B [113]. Therefore, new therapeutic strategies should be optimized for cell‐specific mechanisms, targeting the proliferation of tumor cells and strategies that accelerate or enhance the death signal. This approach could increase the success rate of clinical anti‐mitotic therapy when used to target senescent or chromosomally abnormal stressed cells.

## 8 Conclusions

Given CENP‐F's pivotal role as a centromere protein in somatic cell mitosis, it is imperative to delve into its activity and function in relation to tumor initiation and progression. Accumulating evidence indicates that the deletion of CENP‐F is linked to various mitotic abnormalities. Notably, CENP‐F not only fuels the malignant advancement of tumors but may also serve as a guiding star in tumor treatment strategies. Advancements in therapeutic mRNA vaccine technology have paved the way for the development of personalized vaccines targeting tumor neoantigens, marking a significant direction in the realm of tumor immunology. These mRNA tumor vaccines can be tailor‐made based on the tumor antigens expressed by cancer cells, thereby eliciting robust anti‐tumor T‐cell or B‐cell responses within the body. Clinical studies have already demonstrated the feasibility of mRNA vaccines in cancer treatment, underscoring their promising clinical application potential for future cancer therapies. In light of this, future clinical trials should focus on patients with surgically resected cancer, aiming to induce a potent immune response and mitigate the risk of cancer recurrence. This can be achieved through the expression of CENP‐F mRNA in patient serum, intravenous administration using lipid nanoparticles, or by combining the vaccine with standard therapy and immune checkpoint inhibitors. However, the precise mechanism underlying CENP‐F's regulation of cancer pathogenesis remains elusive. Researchers have explored the CENP‐F mechanism from various angles, including controlling proliferation and metabolism, regulating apoptotic pathways and metabolic reprogramming, inducing angiogenesis, and activating invasion and metastasis. Yet, deeper research is still required to pinpoint the crucial pathways through which CENP‐F orchestrates carcinogenesis. Such endeavors will undoubtedly uncover novel therapeutic targets and strategies for cancers associated with centromere proteins.

## Author Contributions

**Zitong Wan:** conceptualization (lead), funding acquisition (lead), investigation (lead), methodology (lead), project administration (lead), resources (lead), software (lead), supervision (lead), visualization (lead), writing – original draft (lead), writing – review and editing (equal). **Miaomiao Wen:** conceptualization (equal), investigation (equal), methodology (equal), project administration (equal), supervision (equal), visualization (equal), writing – review and editing (equal). **Chunlong Zheng:** conceptualization (equal), funding acquisition (equal), investigation (equal), methodology (equal), software (equal), supervision (equal), visualization (equal), writing – review and editing (equal). **Ying Sun:** investigation (supporting), methodology (supporting), project administration (supporting), software (supporting), supervision (supporting), writing – review and editing (supporting). **Yinxi Zhou:** investigation (supporting), methodology (supporting), project administration (supporting), software (supporting), supervision (supporting). **Yahui Tian:** conceptualization (supporting), funding acquisition (supporting), investigation (supporting), methodology (supporting), supervision (supporting), writing – review and editing (supporting). **Shaowei Xin:** conceptualization (supporting), methodology (supporting), resources (supporting), software (supporting), supervision (supporting), writing – review and editing (supporting). **Xuejiao Wang:** formal analysis (supporting), investigation (supporting), methodology (supporting), software (supporting). **Xiaohong Ji:** formal analysis (supporting), resources (supporting), supervision (supporting), visualization (supporting). **Jie Yang:** formal analysis (supporting), methodology (supporting), resources (supporting), software (supporting). **Yanlu Xiong:** conceptualization (lead), formal analysis (supporting), funding acquisition (supporting), investigation (supporting), methodology (supporting), project administration (supporting), resources (supporting), supervision (supporting), writing – original draft (lead), writing – review and editing (lead). **Yong Han:** conceptualization (lead), investigation (supporting), methodology (supporting), project administration (supporting), resources (supporting), supervision (supporting), writing – original draft (lead), writing – review and editing (lead).

## Ethics Statement

The authors have nothing to report.

## Conflicts of Interest

The authors declare no conflicts of interest.

## Data Availability

Data sharing is not applicable to this article as no new data were created or analyzed in this study.
